# HAUS5 Is A Potential Prognostic Biomarker With Functional Significance in Breast Cancer

**DOI:** 10.3389/fonc.2022.829777

**Published:** 2022-02-25

**Authors:** Zhijian Huang, Jiasheng Yang, Wenjing Qiu, Jing Huang, Zhirong Chen, Yuanyuan Han, Changsheng Ye

**Affiliations:** ^1^ Breast Center, Nanfang Hospital, Southern Medical University, Guangzhou, China; ^2^ Department of Breast Surgical Oncology, Fujian Medical University Cancer Hospital, Fujian Cancer Hospital, Fuzhou, China; ^3^ School of Electrical and Information Engineering, Anhui University of Technology, Maanshan, China; ^4^ Department of Pharmacy, Fujian Medical University Cancer Hospital, Fujian Cancer Hospital, Fuzhou, China; ^5^ Biomedical Research Center of South China, Fujian Normal University, Fuzhou, China; ^6^ Institute of Medical Biology, Chinese Academy of Medical Sciences and Peking Union Medical College, Kunming, China

**Keywords:** HAUS5, bioinformatics, breast cancer, biomarker, immune infiltration, prognosis

## Abstract

**Background:**

Breast cancer (BRCA) has become the most frequently appearing, lethal, and aggressive cancer with increasing morbidity and mortality. Previously, it was discovered that the HAUS5 protein is involved in centrosome integrity, spindle assembly, and the completion of the cytoplasmic division process during mitosis. By encouraging chromosome misdivision and aneuploidy, HAUS5 has the potential to cause cancer. The significance of HAUS5 in BRCA and the relationship between its expression and clinical outcomes or immune infiltration remains unclear.

**Methods:**

Pan-cancer was analyzed by TIMER2 web and the expression differential of HAUS5 was discovered. The prognostic value of HAUS5 for BRCA was evaluated with KM plotter and confirmed with Gene Expression Omnibus (GEO) dataset. Following that, we looked at the relationship between the high and low expression groups of HAUS5 and breast cancer clinical indications. Signaling pathways linked to HAUS5 expression were discovered using Gene Set Enrichment Analysis (GSEA). The relative immune cell infiltrations of each sample were assessed using the CIBERSORT algorithm and ESTIMATE method. We evaluated the Tumor Mutation Burden (TMB) value between the two sets of samples with high and low HAUS5 expression, as well as the differences in gene mutations between the two groups. The proliferation changes of BRCA cells after knockdown of HAUS5 were evaluated by fluorescence cell counting and colony formation assay.

**Result:**

HAUS5 is strongly expressed in most malignancies, and distinct associations exist between HAUS5 and prognosis in BRCA patients. Upregulated HAUS5 was associated with poor clinicopathological characteristics such as tumor T stage, ER, PR, and HER2 status. mitotic prometaphase, primary immunodeficiency, DNA replication, cell cycle related signaling pathways were all enriched in the presence of elevated HAUS5 expression, according to GSEA analysis. The BRCA microenvironment’s core gene, HAUS5, was shown to be related with invading immune cell subtypes and tumor cell stemness. TMB in the HAUS5-low expression group was significantly higher than that in the high expression group. The mutation frequency of 15 genes was substantially different in the high expression group compared to the low expression group. BRCA cells’ capacity to proliferate was decreased when HAUS5 was knocked down.

**Conclusion:**

These findings show that HAUS5 is a positive regulator of BRCA progression that contributes to BRCA cells proliferation. As a result, HAUS5 might be a novel prognostic indicator and therapeutic target for BRCA patients.

## Introduction

Female breast cancer (BRCA) has surpassed lung cancer as the most frequent cancer, with an estimated 2.26 million new cases (11.7%) (1), and is the leading cause of cancer deaths among women globally (2). Because of advancements in early screening and the development of anti-cancer strategies, BRCA therapy has vastly improved. however, the recurrence and metastasis rate of BRCA remains high (3, 4). Due to tumor heterogeneity and complexity, existing biomarkers are limited in predicting BRCA prognosis (5). Therefore, it is urgent to explore new molecular biomarkers as prognostic indicators in the field of clinical research to enhance prognosis and guide individualized treatment strategies (6).

HAUS5 (Augmin like complex subunit 5), also known as Dgt5 or KIAA0841, is one of the eight subunits of the augmin complex (7). During cell division, it mainly participates in spindle assembly, centrosome integrity, and cytoplasmic division (8). Abnormal expression of HAUS5 can induce microtubule fragmentation of centrosome and increment of centrosome volume, leading to chromosome dislocation and functional defects of bipolar spindle (9), which in turn may induce tumor formation. It suggests that HAUS5 may be involved in the onset and progression of breast cancer. However, there are currently inadequate research s on the influence of HAUS5 on breast cancer. As a result, greater research into the relevance and function of HAUS5 in BRCA pathogenesis is required.

In the study, we investigated the link between HAUS5 mRNA expression and BRCA clinical characteristics, as well as the possible role of HAUS5 in BRCA patients’ prognosis and immune regulations. *In vitro*, the impact of HAUS5 on BRCA cells was investigated further. Our study highlights the potential carcinogenic role of HAUS5 in breast cancer. Targeting HAUS5 may be a promising prognostic and chemotherapy target in the future.

## Method

### Datasets and Data Preprocessing

TCGA data breast cancer expression profile, mutation, and clinical data were all downloaded using the function provided by TCGAbiolinks (10), which contained a total of 1092 samples, including 113 normal samples and 979 breast cancer samples. Cases with insufficient or missing data were deleted from subsequent data processing. BRCA patients were classified into low- and high-HAUS5 expression groups according to the median HAUS5 expression value. GSE21653 was downloaded from the GEO database and validated for survival analyses (11).

Data of protein interaction networks are collected from STRING databases. KEGG channel information from GSEA database (https://www.gsea-KEGG.org/gsea/index.jsp). The oncogene list was collected from the COSMIC (12) and OncKB (13) database, the immune-related gene list was collected from IMMPORT (14) database, and the gene information related to breast cancer was collected from CTD (15) database.

### GO Enrichment Analysis

Pan cancer was analyzed by TIMER2 (tumor immune estimation resource, version 2) web (http://timer.cistrome.org/) and observed the expression difference of HAUS5 between tumor and adjacent normal tissues for the different tumors or specific tumor subtypes of the TCGA project. The protein expression of HAUS5 was obtained from the human protein atlas (HPA) (http://www.proteinatlas.org/) database.

### Survival Analysis

To explore whether the expression of the HAUS5 gene is related to the prognosis of breast cancer, we divided the cancer samples into two groups with high and low expression according to the median expression value of HAUS5 and then drew the K-M curve. Both overall survival and breast cancer- specific survival were used as endpoints. The prognostic value of HAUS5 for BRCA was evaluated with KM plotter (https://kmplot.com/analysis/).

### Demographic and Clinical Variables

The relationship between HAUS5 expression and clinical characteristics, including tumor status, lymph node status, distant metastasis, pathologic stage, histological type, PR status, ER status, HER2, and age was analyzed by using χ2 statistics.

### Function and Pathway Analysis by Gene Set Enrichment Analysis (GSEA)

Differentially expressed genes (DEGs) between Low- and high- HAUS5 expression groups were identified by using the DESeq2 R package (version 1.26.0). In this study, GSEA was performed using the ggplot2 R package (v 3.3.3) to demonstrate the significant functions and pathways between the two groups. The expression level of HAUS5 was used as a phenotype label. An adjusted p-value < 0.05, normalized enrichment score (|NES|) > 1, and false discovery rate (FDR) < 0.25 were considered as significant difference.

### TIMER Database Analysis

To explore whether the expression of HAUS5 is related to the tumor immune microenvironment, we first used the ESTIMATE algorithm to estimate the content of stromal cells and immune cells in tumor samples. Secondly, CIBERSORT algorithm was used to evaluate the invasion level of 22 immune cells in the sample and to evaluate the difference in the invasion level of each immune cell between the high and low expression groups (http://timer.cistrome.org/) (16).

### TMB and Mutation Analysis

The mutation data of HAUS5 was obtained from the cBioPortal (https://www.cbioportal.org/) web platform. In the study, we explored the genomic profiles of HAUS5 with a z-score threshold ± 1.5. TMB was obtained by calculating the number of tumor mutations per Mb in each sample (17), and the TMB threshold is 10 mut/Mb. The relationship between HAUS5 and TMB was analyzed. After comparing the TMB value between the two groups with high and low expression of HAUS5, we further analyzed the differences of gene mutations between the two groups of samples.

### PPI Network and KEGG/GO Analysis

To further explore the functional role of HAUS5 in cancer, we collected 100 genes directly interacting with HAUS5 from five protein-interaction network databases: STRING (18) Mentha (19), BioGRID (20), HPRD (21), and IntAct (22). At the same time, to show the function of the HAUS5 gene more directly, we collected the list of genes related to breast cancer from the CTD database, the list of oncogenes from the COSMIC and OncKB database, and the list of immune-related genes from IMMPORT database. Finally, all the information was annotated into the interaction network, and Cytoscape (23) was used to visualize the gene interaction network. To further explore the functions of this interaction network module dominated by HAUS5, we conducted go and KEGG enrichment analysis on these genes.

### Cell Culture

The human breast cancer cell line (MDA MB 231 and HCC1937) was obtained from Shanghai Cell Bank (Shanghai, China) and cells were cultured in DMEM high glucose and supplemented with 10% fetal bovine serum (HyClone), 100 units/ml penicillin (HyClone) and 100 μg/ml streptomycin (HyClone) at 37°C in a humidified chamber with 5% CO2.

### RNA Interference and Cell Counting Assay

Lentiviral vector (Shanghai GeneChem Co., Ltd., Shanghai, China). Subsequently, 293T cells were co-transfected with lentiviral vector carrying the shHAUS5 or negative control shRNA and packaged plasmids. The lentivirus were then harvested and the virus titer was determined. In addition, lentiviral vectors carried the green fluorescent protein (GFP) gene to label tumor cells. After 24 h of transfection, the medium was changed and cultured at 37°C for another 72 h. The images of infected cells were observed under a phase-contrast and fluorescence microscope. Logarithmic growth phase HCC1937 and MDA-MB 231 cells infected with lentivirus carrying shHAUS5 or shCtrl, were seeded into plates and incubated at 37°C with 5% CO2 for up to 5 days. The cells were then counted. The experiments were performed in triplicate and repeated at least three times independently.

### Colony Formation Assay

HCC1937 and MDA-MB 231cells (1,000 cells/well) were seeded into 6-well plates. After adhesion cells were cultured in DMEM at 37°C for 14 days. During this period, the cells were washed with PBS every 3 days, fixed with paraformaldehyde (4%, 15 minutes), and stained with crystal violet (0.1%, 15 minutes) and the numbers of colonies with > 50 cells were counted. All experiments were repeated at least three times.

### Statistical Analysis

We described the baseline characteristics of the patients and treatment using summary statistics. Differences between qualitative variables and continuous variables were analyzed using χ2 statistics, t-test, and analysis of variance (ANOVA), respectively. Survival was estimated using the Kaplan–Meier method and compared between the different groups using the log-rank test. All the tests above were 2-tailed, and a p-value of less than 0.05 was considered statistically significant.

## Results

### HAUS5 Was Up-Regulated in Pan-Cancer and Has Prognostic Value in BRCA

**Figure 1:**
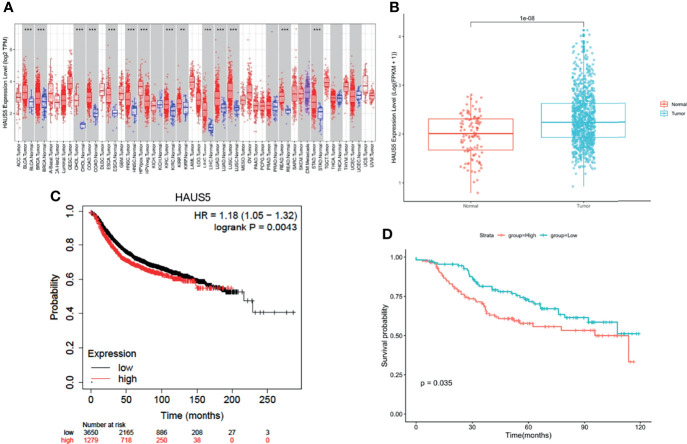
HAUS5 was up-regulated in pan-cancer and has prognostic value in BRCA. **(A)** The expression of HAUS5 gene in pan-cancer from TCGA data. **(B)** Expression of HAUS5 in BRCA and paracancer. **(C)** OS between the high-low expression groups of HAUS5 gene in KM database and **(D)** DFS in GSE21653. **p value < 0.01; ***p value < 0.001. OS, overall survival; DFS, disease-free survival.

To explore the possible role of HAUS5 in carcinogenesis, we first analyzed the expression of the HAUS5 gene in 37 human cancers of TCGA using the TIMER database. As shown in **Figure 1A**, HAUS5 was significantly overexpressed in 14 cancers compared with normal samples, including BLCA, BRCA, CHOL, COAD, ESCA, HNSC, KIRC, KIRP, LIHC, LUAD, LUSC, PRAD, READ, and STAD. We compared the expression of the HAUS5 gene in cancer and normal samples, and it was illustrated in the boxplot in **Figure 1B** that HAUS5 is significantly overexpressed in cancers. The prognostic value of HAUS5 for BRCA was evaluated with KM plotter (**Figure 1C**) and confirmed with GSE21653 (**Figure 1D**). There was a significant difference in OS and DFS between the high-low expression groups of the HAUS5 gene, indicating that there was a correlation between the expression of this gene and the prognosis of patients.

### Clinical Correlation of HAUS5 in Breast Cancer

Subsequently, we compared the correlation between the high and low expression group of HAUS5 and the clinical indicators of breast cancer. As can be seen from **Table 1**, there were significant differences in tumor T stage, ER, PR and HER2 status between the high and low expression groups. These results also indicate the potential value of HAUS5 for clinical staging.

**Table 1:** Correlation between HAUS5 expression and clinical features.

Variables	n=978	HAUS5		p value (Chi-squared test)
		High (n=489)	Low (n=489)	
Age (years)	978			0.61
<50	256	132 (26.99%)	124 (25.36%)	
≥50	722	357 (73.01%)	365 (74.64)	
Tumor status (T)				0.01777*
T1	250	106 (21.68%)	144 (29.45%)	
T2	566	300 (61.35%)	266 (54.40%)	
T3	126	68 (13.91%)	58 (11.86%)	
T4	33	13 (2.66%)	20 (4.09%)	
unknown	3	2 (0.41%)	1 (0.20%)	
Lymph node status (N)				0.293
N0	469	246 (50.31%)	223 (45.60%)	
N1	312	156 (31.90%)	156 (31.90%)	
N2	108	44 (9%)	64 (13.09%)	
N3	72	35 (7.16%)	37 (7.57%)	
unknown	17	8 (1.64%)	9 (1.84%)	
Distant metastasis (M)				
M0	804	390 (79.75%)	414 (84.66%)	0.131
M1	20	11 (2.25%)	9 (1.84%)	
unknown	154	88 (18%)	66 (13.5)	
Cancer stage				0.139
I	161	73 (14.93%)	88 (18%)	
II	554	296 (60.53%)	258 (52.76%)	
III	223	103 (21.06%)	120 (24.54%)	
IV	18	9 (1.84%)	9 (1.84)	
unknown	22	8 (1.64%)	14 (2.86%)	
ER Status				5.83E-05***
Negative	216	135 (27.61%)	81 (16.56%)	
Positive	725	338 (69.12%)	387 (79.14%)	
unknown	37	16 (3.27%)	21 (4.30%)	
PR Status				0.001551**
Negative	312	180 (36.81%)	132 (26.99%)	
Positive	626	291 (59.51%)	335 (68.51%)	
unknown	40	18 (3.68%)	22 (4.5%)	
HER2 Status				0.0007661****
Negative	503	254 (51.94%)	249 (50.92%)	
Positive	141	48 (9.82%)	93 (19.02%)	
unknown	334	187 (38.24%)	147 (30.06%)	

*P <= 0.05, **P <= 0.01, ***P <= 0.001, ****P <= 0.0001.

ER, estrogen receptor; PR, progesterone receptor; HER2, human epidermal growth factor receptor 2; TNM, tumor, node, metastasis.

### Gene Set Enrichment Analysis (GSEA)

**Figure 2:**
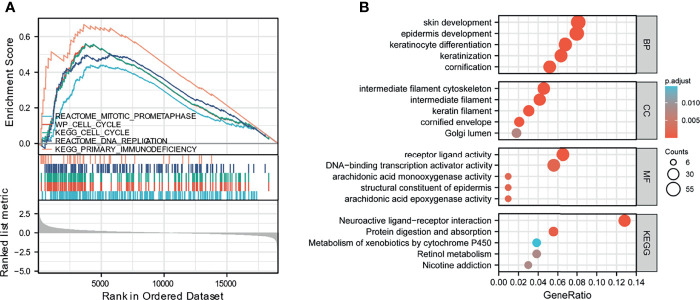
GSEA analysis results. **(A)** Enrichment of genes in the representative pathways by GSEA function analysis. **(B)** GO and KEGG analysis of DEGs in low- and high- HAUS5 expression samples.

To elucidate the biological functions of HAUS5, we analyzed the DEGs between the low- and high- expression HAUS5 groups based on the median HAUS5 expression value. We also performed a GSEA pathway analysis (**Supplementary Table 1**). The results showed that high HAUS5 expression was mainly enriched in mitotic prometaphase, primary immunodeficiency, DNA replication, cell cycle related signaling pathways (**Figure 2A**). At the same time, we completed the GO analysis (**Supplementary Table 2**). BP terms were associated with development, including “skin development”, “epidermis development”, “keratinocyte differentiation”, “keratinization”, and “cornification”. The top 5 CC terms were cell cytoskeleton related, such as “intermediate filament cytoskeleton”, “intermediate filament”, “keratin filament”, “cornified envelope”, and “Golgi lumen”. The top 5 MF terms were associated with “receptor ligand activity”, “transcription activator activity”, “arachidonic acid monooxygenase activity”, “structural constituent of the epidermis”, and “arachidonic acid epoxygenase activity” (**Figure 2B**).

### Analysis of HAUS5 Expression, Copy Number and Related Immune Microenvironment

**Figure 3:**
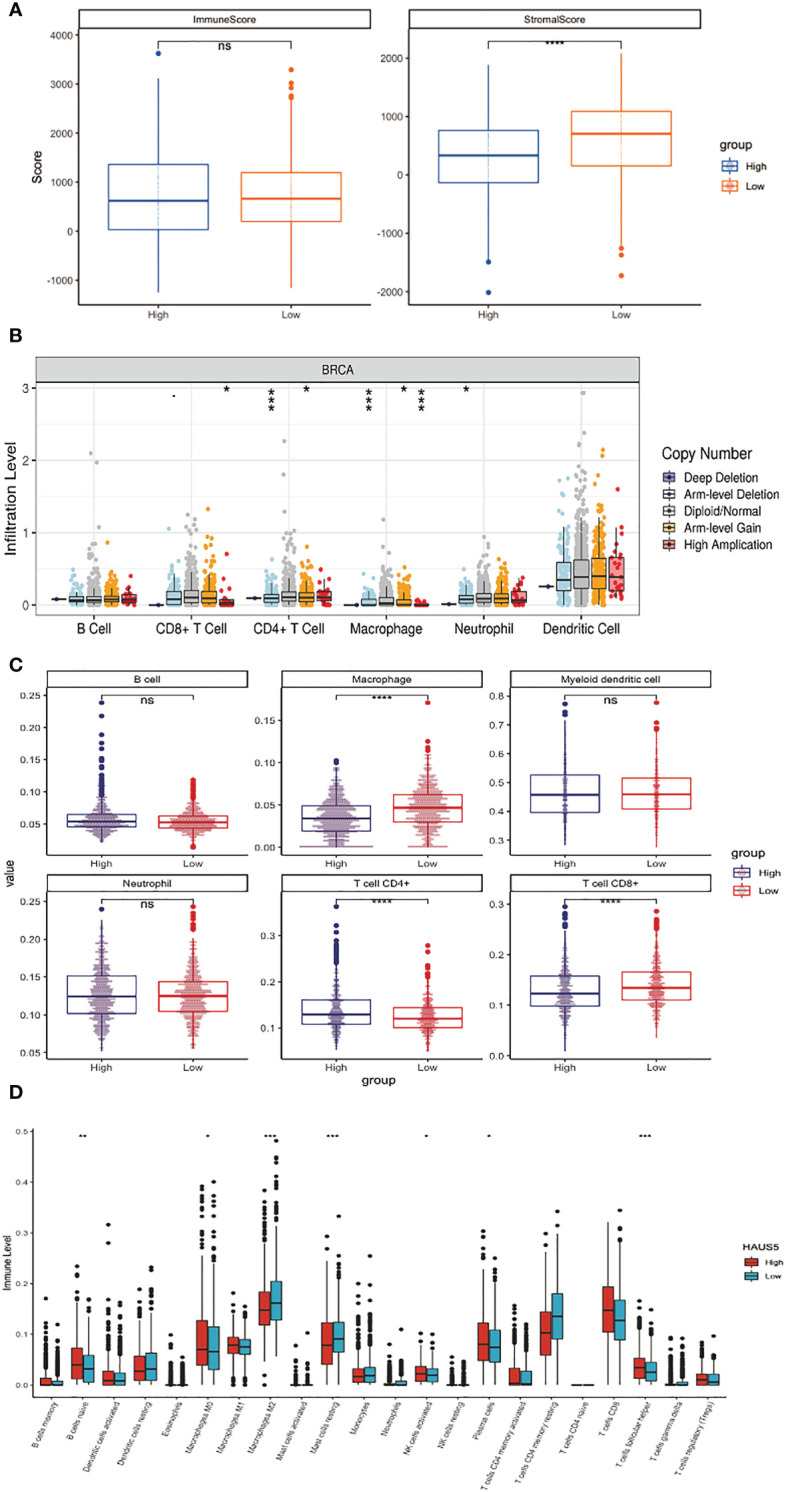
Correlation analysis between HAUS5 and immune microenvironment. **(A)** The correlation between high and low expression of HAUS5 and ESTIMATE score. **(B)** The relationship between copy number variation of HAUS5 gene and immune cell infiltration level. **(C, D)** The relationship between high and low expression of HAUS5 and the level of immune cell infiltration. *p value < 0.05; **p value < 0.01; ***p value < 0.001; ****p value < 0.0001; ns, no sense.

In many cancers, the mRNA expression level of HAUS5 was significantly increased, and the tumor tissue contains not only tumor cells, but also a variety of different types of cells, such as stromal cells, fibroblasts, and immune cells, which together constitute the tumor microenvironment. As shown in **Figure 3A**, the stromal cell score in the group with high HAUS5 expression was significantly lower than that in the group with low HAUS5 expression (P<0.001). Many cancer studies had confirmed that stromal cells play an important role in the development, metastasis and drug resistance of tumors (24). However, there was no difference in immune scores between the high and low expression groups, which may be due to the different composition proportions of different immune cells between the two groups, leading to the insignificant difference. Therefore, in order to further explore the relationship between HAUS5 and tumor immune cells, we used different immune invasion algorithms to calculate the invasion levels of different immune cells.

**Figure 3B** shows that the infiltration levels of CD8+ T Cell, CD4+ T Cell, Macrophage and Neutrophil change with the change of HAUS5 copy number. We had also constructed a stacked bar chart in the Supplementary **Figure 1**, which showed the different relative proportions somatic Copy Number Alteration (sCNA) status of HAUS5 in all TCGA cancer types. As can be seen from **Figure 3C**, Macrophage, CD4+ T Cell and CD8+ T Cell showed significant difference in immune infiltration levels among HAUS5 expression groups (P<0.001). However, there was no difference between the B cell, Myeloid Dendritic cell, and Neutrophil, which confirmed our above conjecture.

According to the **Figure 3D** boxplot, B cells naive, Macrophages M0, Macrophages M2, Mast cells resting, NK cells activated, Plasma cells, and T cells follicular helper, the infiltration levels of the seven kinds of immune cells were significantly different between the high and low expression groups.

The above results have confirmed the correlation between the HASU5 gene and stromal cells and immune cells in the immune microenvironment. The expression or copy number variation of HAUS5 gene will affect the tumor microenvironment and may play an important role in the occurrence, development, metastasis, and immune response of cancer.

### TMB Analysis

**Figure 4:**
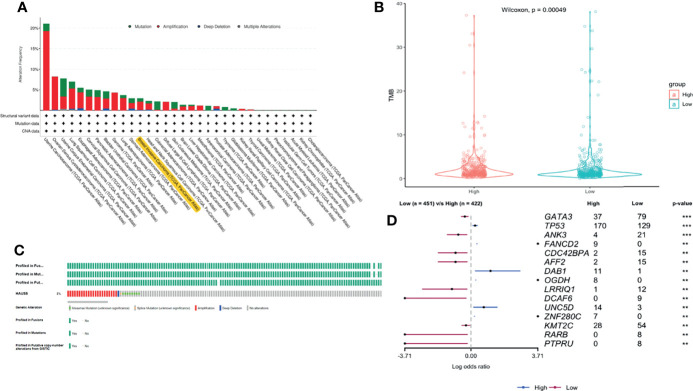
The gene alteration of HAUS5 and TMB difference between high and low expression groups of HAUS5. The gene alteration of HAUS5 in pan-cancer **(A)** and especially in BRCA **(C)**, TMB analysis of HAUS5 in BRCA **(B)**, Mutation difference of HAUS5 expression groups **(D)**. **p<=0.01, ***p<=0.001.

We used cBioPortal (https://www.cbioportal.org/) to analyze the HAUS5 expression and its mutation in BRCA. As was shown in **Figures 4A, C**, a relatively high mutation rate of HAUS5 was observed in BRCA patients. In the 1084 sequenced BRCA patients, the genetic alteration was found in 32 BRCA patients and the mutation rate was 3%.

Although PD-1/PD-L1 immune checkpoint blocking therapy has been approved for the treatment of a variety of malignancies, only a small number of patients benefit from treatment (25). How to screen out patients who can benefit from the massive number of patients is particularly important. TMB is an emerging biomarker for the prediction of immune efficacy. Due to the High mutation load of a tumor, patients with TMB High produce a lot of antigens in the body and have a more adequate response to immunotherapy, with greater clinical benefits. Therefore, we continue to analyze the relationship between HAUS5 and TMB. As can be seen from the violin diagram in **Figure 4B** TMB in the low expression group was significantly higher than that in the high expression group (P<0.001), which further emphasized the correlation between the HAUS5 gene and immunity.

15 genes with the significant difference in mutation frequency was shown in the forest map (**Figure 4D** P<0.01). The mutation frequency of TP53, FANCD2, DAB1 and UNC5D genes in the high expression group was significantly higher than that in the low expression group. The mutation frequency of GATA3, ANK3, CDC42BPA and AFF2 genes in the low expression group was significantly higher than that in the high expression group. CDC42BPA binds to the cell cycle division protein CDC42 as a downstream effector (26). CDC42 can play an important role as a gene for targeted therapy of breast cancer (27, 28). GATA3 is a member of the transcription factor GATA family and can be used as a molecular marker of breast cancer (29). Somatic mutation of GATA3 is associated with clinicopathological features and expression of TCGA in breast cancer patients (30). FANCD2 (31), DAB1 (32) and ANK3 (33) are all supported by literature to be associated with breast cancer.

### HAUS5 Interaction Network and Functional Analysis

**Figure 5:**
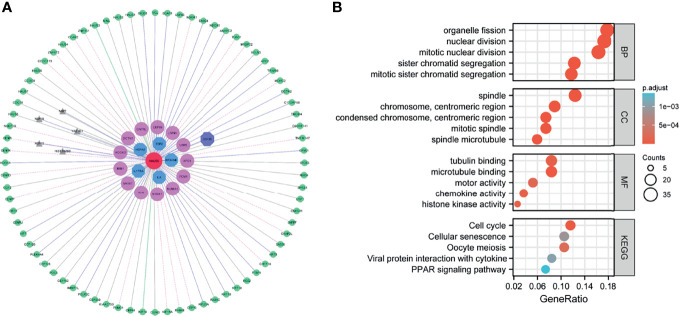
HAUS5 interaction network and functional enrichment analysis. HAUS5-dominated interaction network **(A)**, functional enrichment of HAUS5-dominated interactive network modules **(B)**.

Among the 100 genes directly interacting with HAUS5(**Supplementary Table 3**), gray refers to the genes not annotated by the above database, while the genes in other colors are all breast cancer-related genes, including 14 pink genes, 4 blue genes, and purple genes, which are both cancer genes and immune-related genes in **Figure 5A**. More or less, these key genes have been proved to be closely related to breast cancer by relevant studies. For example, studies have shown that GSK3B gene is a potential drug target of triple negative breast cancer, which can regulate epithelial mesenchymal transformation and tumor stem cell characteristics (34). This result also indicates that HAUS5 may play an important role in the occurrence and development of cancer or the immune response of tumor, and can be used as a potential target.

We performed a GO analysis (**Supplementary Table 4**), and **Figure 5B** shows the top 5 enrichment results respectively. Many of these biological processes are closely related to cell division, for example, organelle fission, nuclear division, sister chromatid segregation. The molecular function enrichment involved in chemotaxes, such as tubulin binding, microtubule binding, motor activity, chemokine activity and histone kinase activity. The cellular component enrichment involved in spindle and centromeric region. In addition, pathway information collected in KEGG database was used for functional enrichment, with a total of 5 pathways shown in **Figure 5B** including cell cycle, cellular senescence, oocyte meiosis, PPAR signaling pathway. These pathways are also closely involved in cell cycle regulation. These results suggest that this functional module may play an important role in cell proliferation, division, and cell cycle regulation.

### Knockdown of HAUS5 Inhibits BRCA Cells Progression *In Vitro* and the Protein Expression of HAUS5 in HPA

**Figure 6:**
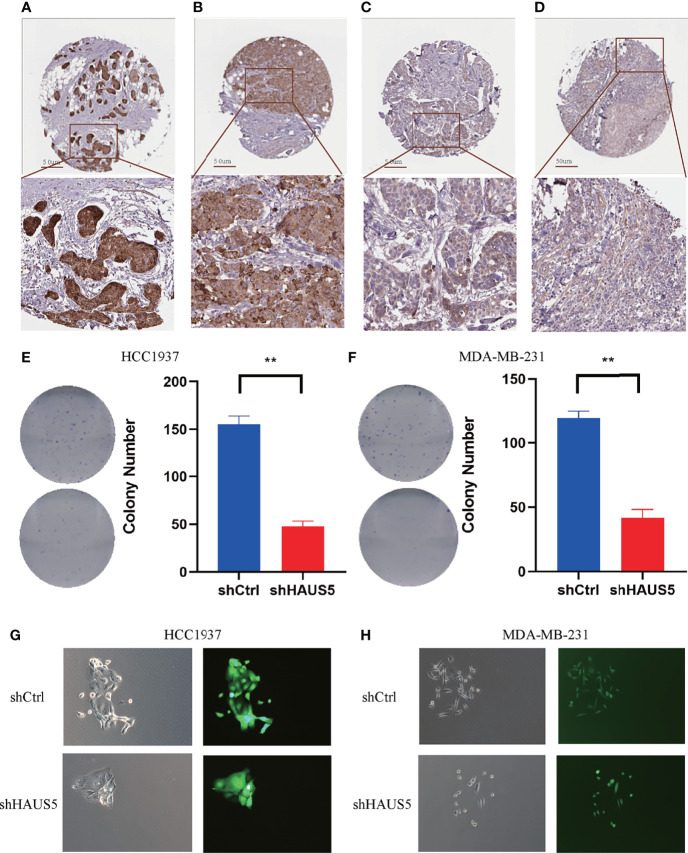
The protein expression of HAUS5 in BRCA and the effects of HAUS5 knockdown with colong formation assay. The protein expression of HAUS5 was obtained from the Human Protein Atlas, High **(A)**, median **(B)**, low **(C)**, and not detected **(D)**. Colong formation assay showed the proliferation of HAUS5 knocked down in HCC1937 **(E)** and MDA-MB-231 **(F)**. Fluorescence microscopy demonstrated the expression of GFP in a transfected clone colony in HCC1937 **(G)** and MDA-MB-231 **(H)**. **p value < 0.01; Ctrl, negative control; sh, short hairpin RNA.

**Figure 7:**
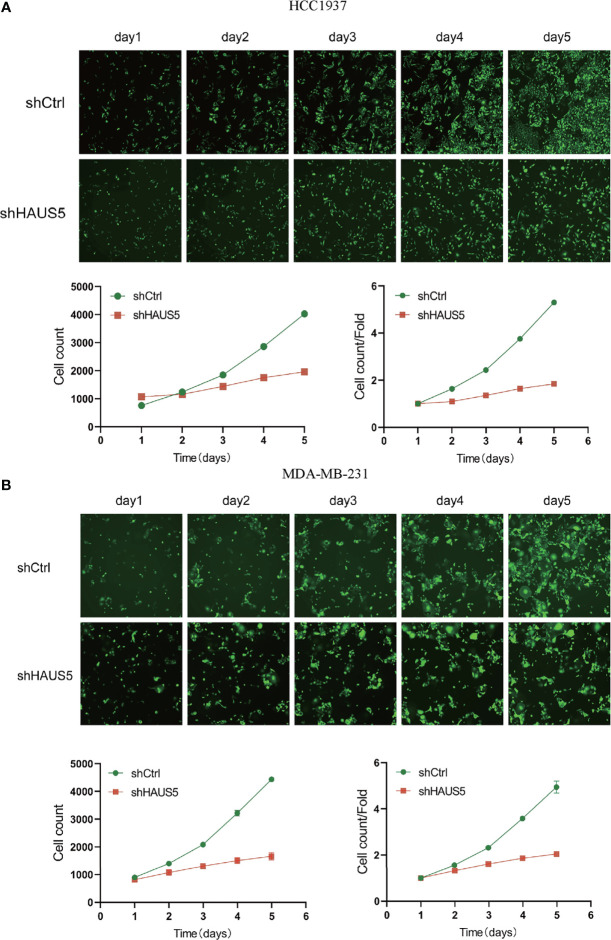
The effects of HAUS5 knockdown with tumor cell counting assay. The knockdown of HAUS5 by shHAUS5 and shCtrl in HCC1937 cells **(A)** and MDA−MB−231 cells **(B)** using counting assay. The data were expressed as the means ± standard deviation.

Immunohistochemistry-based antibody-specific staining scores in breast tumors were obtained from the Human Protein Atlas, in which tumor-specific staining was divided into four grades: high, medium, low and undetected (**Figures 6A–D**). To explore the oncogenic role of overexpression HAUS5 in BRCA, HCC1937 and MDA-MB-231 cells were transfected with sh-HAUS5 or sh-NC and performed colony formation assay (**Figures 6E–H**) and fluorescence cell counting (**Figure 7**). The findings showed that knockdown of HAUS5 significantly suppressed the proliferation ability of BRCA cell lines.

## Discussion

In this study, we first found that HAUS5 mRNA overexpressed in breast cancer. Meanwhile, HAUS5 mRNA expression also plays a huge role in the diagnosis of BRCA. In addition, by mining the clinical value of HAUS5 mRNA expression in different clinical variables, including tumor status, lymph node status, distant metastasis, pathologic stage, histological type, PR status, ER status, HER2. Besides, HAUS5 mRNA expression predicted poor prognosis and served as an independent prognostic factor for overall survival.

To further analyze the function of HAUS5, we conducted immune microenvironment analysis and found that there was a significant difference in the proportion of stromal cells between high and low expression groups. Although there was no difference in immune score, the infiltration level of different cells was significantly correlated with the copy number of HAUS5 from the point of view of immune cell infiltration level. The expression of HAUS5 also affects the infiltration level of different immune cells. Moreover, there were significant differences in TMB between high and low expression groups. TMB has been proved to be related to the response rate of the PD-1 antibodies in many previous studies (35). The higher the TMB is, the higher the PD-1 antibody response rate (36). This indicates that the high expression of this gene may cause breast cancer patients to have no immune response to the PD-1 antibody, which may be a prognostic marker of the PD-1 antibody. In addition, the copy number variation of the gene is also related to the infiltrating immune cells in the tumor tissue (37). There are fewer infiltrating immune cells in the tumor tissue with increased or decreased copy number, indicating that the copy number variation of the gene may affect the immune cell infiltration, which needs to be further studied. On the other hand, the gene may affect the expression of chemokine receptors in tumor tissue. Therefore, the different changes of immune cell infiltration caused by affecting the expression of chemokine receptors may be one of the mechanisms of copy number variation of the gene affecting immune cell infiltration. The lower the transcription level of the gene, the higher the infiltration of CD8+T cells. It may be that the low expression of the gene is related to the increase of TMB and produces more new tumor antigens. Therefore, it can recruit newer antigen specific CD8+T cells to infiltrate into tumor tissues and produce killing effects.

Subsequently, we analyzed the difference in mutation frequency between the high and low expression groups, and found that the mutation frequency of TP53 and other genes in the high expression group was significantly higher than that in the low expression group, while the mutation frequency of GATA3 and other genes in the low expression group was higher. Therefore, the expression level of HAUS5 is related to the level of immunity and mutation of tumor cells.

To further analyze the function of HAUS5, we collected 100 genes directly interacting with HAUS5 from the protein interaction network database, and jointly constructed an interaction network module dominated by HAUS5 genes. From the interaction network diagram, we can see that several oncogenes and immune-related genes directly interact with HAUS5, and many genes have been supported to be related to breast cancer, indicating that the HAUS5 gene we studied may have a very important function in breast cancer. Subsequently, we conducted GO and KEGG enrichment to explore the function of this module. From the results of our enrichment analysis, it can be easily seen that this module plays an important role in the regulation of cell cycle.

In *in vitro* study, we also investigated the role of HAUS5 in the proliferation of BR cells. We found that the knockout of HAUS5 suppressed the growth of breast cells by fluorescence cell counting and colony formation assay. However, there is some limitation in this study. For example, it might be interesting to study HAUS5 in breast cancer cells at the single cell level (38, 39). In addition, identifying drugs targeting HAUS5 might push the results into clinical practice (40). Finally, we need more *in vitro* and *in vivo* experiment to validated the exact role of HAUS5 in BRCA and how does it work.

## Conclusion

HAUS5 can be used as a potential marker and deserves further analysis to explore its role in the process of tumor occurrence, development, metastasis, and differentiation associated with immune infiltration from the experimental level.

## Data Availability Statement

The original contributions presented in the study are included in the article/**Supplementary Material**. Further inquiries can be directed to the corresponding authors.

## Author Contributions

CY and YH conceived and designed the study. ZH, JY, WQ, and JH performed the experiments. ZH and ZC analyzed the data. ZH and JH wrote the manuscript. All authors have read and approved this manuscript.

## Funding

This study was supported by the Natural Science Foundation of Fujian Province (No. 2020J011112). Joint Funds for the Innovation of Science and Technology, Fujian Province (Grant number: 2020Y9039) and Yunnan Fundamental Research Projects (grant NO. 202001AT070145).

## Conflict of Interest

The authors declare that the research was conducted in the absence of any commercial or financial relationships that could be construed as a potential conflict of interest.

## Publisher’s Note

All claims expressed in this article are solely those of the authors and do not necessarily represent those of their affiliated organizations, or those of the publisher, the editors and the reviewers. Any product that may be evaluated in this article, or claim that may be made by its manufacturer, is not guaranteed or endorsed by the publisher.

## References

[1] Sung H Ferlay J Siegel RL Laversanne M Soerjomataram I Jemal A. Global Cancer Statistics 2020: GLOBOCAN Estimates of Incidence and Mortality Worldwide for 36 Cancers in 185 Countries. CA Cancer J Clin (2021) 71(3):209–49. doi: 10.3322/caac.21660 33538338

[2] Siegel RL Miller KD Jemal A. Cancer Statistics 2019. CA Cancer J Clin (2019) 69(1):7–34. doi: 10.3322/caac.21551 30620402

[3] Waks AG Winer EP. Breast Cancer Treatment: A Review. JAMA (2019) 321(3):288–300. doi: 10.1001/jama.2018.19323 30667505

[4] Liu H Qiu C Wang B Bing P Tian G Zhang X. Evaluating DNA Methylation, Gene Expression, Somatic Mutation, and Their Combinations in Inferring Tumor Tissue-Of-Origin. Front Cell Dev Biol (2021) 9:619330. doi: 10.3389/fcell.2021.619330 34012960 PMC8126648

[5] Hunter NB Kilgore MR Davidson NE. The Long and Winding Road for Breast Cancer Biomarkers to Reach Clinical Utility. Clin Cancer Res (2020) 26(21):5543–5. doi: 10.1158/1078-0432.CCR-20-2451 PMC8791049 32859655

[6] Zhang Y Xiang J Tang L Li J Lu Q Tian G. Identifying Breast Cancer-Related Genes Based on a Novel Computational Framework Involving KEGG Pathways and PPI Network Modularity. Front Genet (2021) 12:596794. doi: 10.3389/fgene.2021.596794 34484285 PMC8415302

[7] Lawo S Bashkurov M Mullin M Ferreria MG Kittler R Habermann B. HAUS, the 8-Subunit Human Augmin Complex, Regulates Centrosome and Spindle Integrity. Curr Biol (2009) 19(10):816–26. doi: 10.1016/j.cub.2009.04.033 19427217

[8] Tian J Kong Z. The Role of the Augmin Complex in Establishing Microtubule Arrays. J Exp Bot (2019) 70(12):3035–41. doi: 10.1093/jxb/erz123 30882862

[9] Schweizer N Haren L Dutto I Viais R Lacasa C Merdes A. Sub-Centrosomal Mapping Identifies Augmin-Gammaturc as Part of a Centriole-Stabilizing Scaffold. Nat Commun (2021) 12(1):6042. doi: 10.1038/s41467-021-26252-5 34654813 PMC8519919

[10] Colaprico A Silva TC Olsen C Garofano L Cava C Garolini D. TCGAbiolinks: An R/Bioconductor Package for Integrative Analysis of TCGA Data. Nucleic Acids Res (2016) 44(8):e71. doi: 10.1093/nar/gkv1507 26704973 PMC4856967

[11] Sabatier R Finetti P Adelaide J Guille A Borg JP Chaffanet M. Down-Regulation of ECRG4, a Candidate Tumor Suppressor Gene, in Human Breast Cancer. PLoS One (2011) 6(11):e27656. doi: 10.1371/journal.pone.0027656 22110708 PMC3218004

[12] Forbes SA Bhamra G Bamford S Dawson E Kok C Clements J. The Catalogue of Somatic Mutations in Cancer (COSMIC). Curr Protoc Hum Genet (2008) Chapter 10:Unit 10 11. doi: 10.1002/0471142905.hg1011s57 PMC2705836 18428421

[13] Chakravarty D Gao J Phillips SM Kundra R Zhang H Wang J. OncoKB: A Precision Oncology Knowledge Base. JCO Precis Oncol (2017) 1:1–16. doi: 10.1200/PO.17.00011 PMC5586540 28890946

[14] Bhattacharya S Dunn P Thomas CG Smith B Schaefer H Chen J. ImmPort, Toward Repurposing of Open Access Immunological Assay Data for Translational and Clinical Research. Sci Data (2018) 5:180015. doi: 10.1038/sdata.2018.15 29485622 PMC5827693

[15] Davis AP Grondin CJ Johnson RJ Sciaky D Wiegers J Wiegers TC. Comparative Toxicogenomics Database (CTD): update 2021. Nucleic Acids Res (2021) 49(D1):D1138–43. doi: 10.1093/nar/gkaa891 PMC7779006 33068428

[16] Li T Fu J Zeng Z Cohen D Li J Chen Q. TIMER2.0 for Analysis of Tumor-Infiltrating Immune Cells. Nucleic Acids Res (2020) 48(W1):W509–14. doi: 10.1093/nar/gkaa407 PMC7319575 32442275

[17] Krasniqi E Barchiesi G Pizzuti L Mazzotta M Venuti A Maugeri-Sacca M. Immunotherapy in HER2-Positive Breast Cancer: State of the Art and Future Perspectives. J Hematol Oncol (2019) 12(1):111. doi: 10.1186/s13045-019-0798-2 31665051 PMC6820969

[18] Szklarczyk D Gable AL Nastou KC Lyon D Kirsch R Pyysalo S. The STRING Database in 2021: Customizable Protein-Protein Networks, and Functional Characterization of User-Uploaded Gene/Measurement Sets. Nucleic Acids Res (2021) 49(D1):D605–12. doi: 10.1093/nar/gkaa1074 PMC7779004 33237311

[19] Calderone A Castagnoli L Cesareni G. Mentha: A Resource for Browsing Integrated Protein-Interaction Networks. Nat Methods (2013) 10(8):690–1. doi: 10.1038/nmeth.2561 23900247

[20] Stark C Breitkreutz BJ Reguly T Boucher L Breitkreutz A Tyers M. BioGRID: A General Repository for Interaction Datasets. Nucleic Acids Res (2006) 34(Database issue):D535–9. doi: 10.1093/nar/gkj109 PMC1347471 16381927

[21] Keshava Prasad TS Goel R Kandasamy K Keerthikumar S Kumar S Mathivanan S. Human Protein Reference Database–2009 Update. Nucleic Acids Res (2009) 37(Database issue):D767–72. doi: 10.1093/nar/gkn892 PMC2686490 18988627

[22] Aranda B Achuthan P Alam-Faruque Y Armean I Bridge A Derow C. The IntAct Molecular Interaction Database in 2010. Nucleic Acids Res (2010) 38(Database issue):D525–31. doi: 10.1093/nar/gkp878 PMC2808934 19850723

[23] Shannon P Markiel A Ozier O Baliga NS Wang JT Ramage D. Cytoscape: A Software Environment for Integrated Models of Biomolecular Interaction Networks. Genome Res (2003) 13(11):2498–504. doi: 10.1101/gr.1239303 PMC403769 14597658

[24] Turley SJ Cremasco V Astarita JL. Immunological Hallmarks of Stromal Cells in the Tumour Microenvironment. Nat Rev Immunol (2015) 15(11):669–82. doi: 10.1038/nri3902 26471778

[25] Khair DO Bax HJ Mele S Crescioli S Pellizzari G Khiabany A. Combining Immune Checkpoint Inhibitors: Established and Emerging Targets and Strategies to Improve Outcomes in Melanoma. Front Immunol (2019) 10:453. doi: 10.3389/fimmu.2019.00453 30941125 PMC6435047

[26] Hu HF Xu WW Wang Y Zheng CC Zhang WX Li B. Comparative Proteomics Analysis Identifies Cdc42-Cdc42BPA Signaling as Prognostic Biomarker and Therapeutic Target for Colon Cancer Invasion. J Proteome Res (2018) 17(1):265–75. doi: 10.1021/acs.jproteome.7b00550 29072916

[27] Maldonado MDM Dharmawardhane S. Targeting Rac and Cdc42 GTPases in Cancer. Cancer Res (2018) 78(12):3101–11. doi: 10.1158/0008-5472.CAN-18-0619 PMC6004249 29858187

[28] Zhang Y Li J Lai XN Jiao XQ Xiong JP Xiong LX. Focus on Cdc42 in Breast Cancer: New Insights, Target Therapy Development and Non-Coding RNAs. Cells (2019) 8(2):146. doi: 10.3390/cells8020146 PMC6406589 30754684

[29] Cancer Genome Atlas Network. Comprehensive Molecular Portraits of Human Breast Tumours. Nature (2012) 490(7418):61–70. doi: 10.1038/nature11412 23000897 PMC3465532

[30] Afzaljavan F Sadr AS Savas S Pasdar A. GATA3 Somatic Mutations are Associated With Clinicopathological Features and Expression Profile in TCGA Breast Cancer Patients. Sci Rep (2021) 11(1):1679. doi: 10.1038/s41598-020-80680-9 33462316 PMC7814117

[31] van der Groep P Hoelzel M Buerger H Joenje H de Winter JP van Diest PJ. Loss of Expression of FANCD2 Protein in Sporadic and Hereditary Breast Cancer. Breast Cancer Res Treat (2008) 107(1):41–7. doi: 10.1007/s10549-007-9534-7 PMC2096638 17333336

[32] Cao RJ Li K Xing WY Du S Li Q Zhu XJ. Disabled-1 is Down-Regulated in Clinical Breast Cancer and Regulates Cell Apoptosis Through NF-Kappab/Bcl-2/Caspase-9. J Cell Mol Med (2019) 23(2):1622–7. doi: 10.1111/jcmm.14047 PMC6349202 30484953

[33] Kurozumi S Joseph C Raafat S Sonbul S Kariri Y Alsaeed S. Utility of Ankyrin 3 as a Prognostic Marker in Androgen-Receptor-Positive Breast Cancer. Breast Cancer Res Treat (2019) 176(1):63–73. doi: 10.1007/s10549-019-05216-w 30941650

[34] Vijay GV Zhao N Den Hollander P Toneff MJ Joseph R Pietila M. Gsk3β Regulates Epithelial-Mesenchymal Transition and Cancer Stem Cell Properties in Triple-Negative Breast Cancer. Breast Cancer Res (2019) 21(1):37. doi: 10.1186/s13058-019-1125-0 30845991 PMC6407242

[35] Wang F Wei XL Wang FH Xu N Shen L Dai GH. Safety, Efficacy and Tumor Mutational Burden as a Biomarker of Overall Survival Benefit in Chemo-Refractory Gastric Cancer Treated With Toripalimab, A PD-1 Antibody in Phase Ib/II Clinical Trial NCT02915432. Ann Oncol (2019) 30(9):1479–86. doi: 10.1093/annonc/mdz197 PMC6771223 31236579

[36] Cristescu R Mogg R Ayers M Albright A Murphy E Yearley J. Pan-Tumor Genomic Biomarkers for PD-1 Checkpoint Blockade-Based Immunotherapy. Science (2018) 362(6411):eaar3593. doi: 10.1126/science.aar3593 PMC6718162 30309915

[37] Dai Z Liu P. High Copy Number Variations, Particular Transcription Factors, and Low Immunity Contribute to the Stemness of Prostate Cancer Cells. J Transl Med (2021) 19(1):206. doi: 10.1186/s12967-021-02870-x 33985534 PMC8117623

[38] Xu J Cai L Liao B Zhu W Yang J. CMF-Impute: An Accurate Imputation Tool for Single-Cell RNA-Seq Data. Bioinformatics (2020) 36(10):3139–47. doi: 10.1093/bioinformatics/btaa109 32073612

[39] Zhuang J Cui L Qu T Ren C Yang J. A Streamlined scRNA-Seq Data Analysis Framework Based on Improved Sparse Subspace Clustering. IEEE Access (2021) PP(99):1–1.

[40] Liu C Wei D Xiang J Ren F Huang L Lang J. An Improved Anticancer Drug-Response Prediction Based on an Ensemble Method Integrating Matrix Completion and Ridge Regression. Mol Ther Nucleic Acids (2020) 21:676–86. doi: 10.1016/j.omtn.2020.07.003 PMC7403773 32759058

