# Supplementary figures and images for: HAUS5 Is A Potential Prognostic Biomarker With Functional Significance in Breast Cancer
Source: Front Oncol. 2022 Feb 25;12:829777. doi: 10.3389/fonc.2022.829777 (PMC8913513; doi:10.3389/fonc.2022.829777)

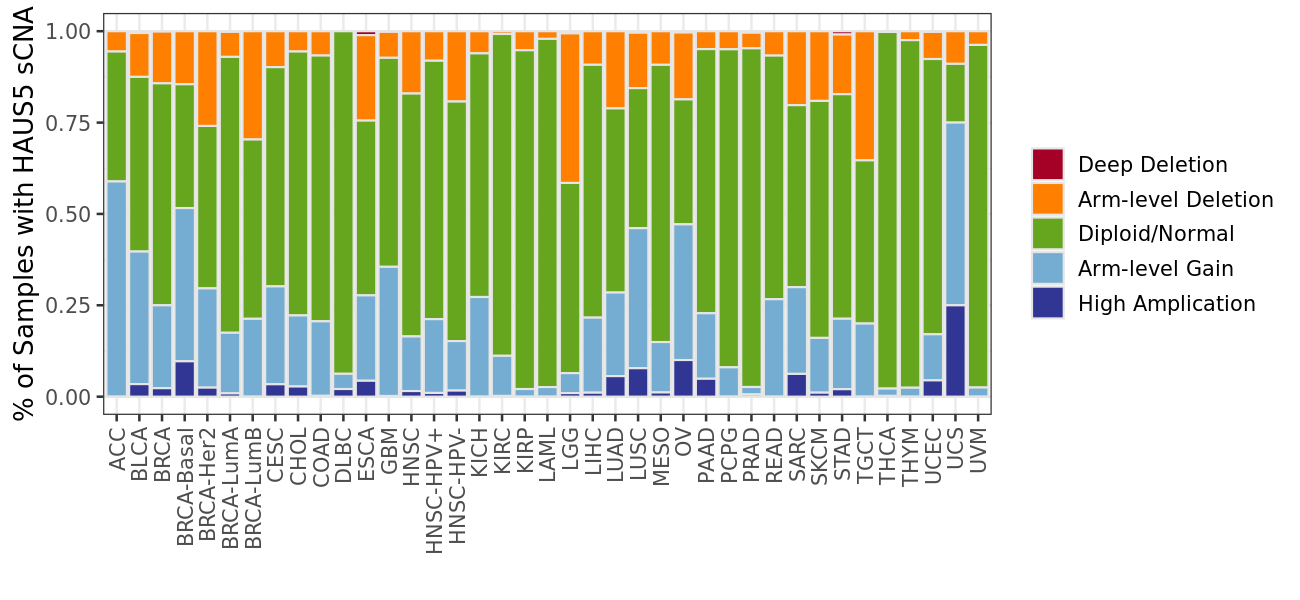

Supplement: Supplementary file 1 [file Image_1.tif]
